# Overexpression of *Hdac6* extends reproductive lifespan in mice

**DOI:** 10.1007/s13238-017-0375-9

**Published:** 2017-03-01

**Authors:** Xiaoxi Zhang, Jiao Yang, Haiying Wang, Renpeng Guo, Yu Yin, Dongdong Zhang, Qian Zhang, Hua Wang, Zhongcheng Zhou, Lingyi Chen, Jun Zhou, Lin Liu

**Affiliations:** State Key Laboratory of Medicinal Chemical Biology, Collaborative Innovation Center for Biotherapy, Department of Cell Biology and Genetics, College of Life Sciences, Nankai University, Tianjin, 300071 China

**Dear Editor,**

Histone deacetylase 6 (Hdac6) was discovered as a deacetylase of α-tubulin and functions in cell migration, immunity and resistance to virus infection *in vitro* (Hubbert et al., 2002; Valenzuela-Fernandez et al., 2008). Overexpression of *Hdac6* enhances resistance to virus infection in embryonic stem (ES) cells and in mice (Wang et al., 2015). Hdac6 also can function to deacetylate protein and is involved in protein ubiquitination and degradation (Seigneurin-Berny et al., 2001; Zhang et al., 2014), and self-clearance of misfolded proteins, promoting autophagy and preventing neurodegeneration (Lee et al., 2010; Pandey et al., 2007). HDAC6 also is implicated in DNA damage response and depletion or inhibition of HDAC6 induces DNA damage and apoptosis (Namdar et al., 2010; Zhang et al., 2014), suggesting that *HDAC6* could be important for DNA repair and integrity maintenance.

DNA damage accumulates with somatic aging and reproductive aging (Titus et al., 2013), and enhanced DNA damage repair can reverse aging (Maynard et al., 2015). Reproductive aging in females mainly results from ovarian senescence, as shown by reduced number and quality of follicles and germ cells. Interestingly, *Hdac6* is highly expressed in germ cell tissues, testis and spermatogenic cells, relative to other somatic tissues, such as liver, heart, muscle, spleen and kidney (Seigneurin-Berny et al., 2001; Zhang et al., 2008), and yet *Hdac6*-deficient mice are viable and fertile, and show normal development and function of testis (Zhang et al., 2008). Initially, we tested whether DNA damage is reduced in *Hdac6* overexpression embryonic stem (ES) cells, which expressed higher Hdac6 protein levels and lower levels of α-acetylated tubulin than did WT ES cells (Wang et al., 2015). Treatment of mouse embryonic fibroblasts (MEFs) with mitomycin C can generate mitotically inactive feeder cells and these cells showed high levels of DNA damage as shown by many γH2AX foci served as positive control (Fig. S1A and 1B). Fewer γH2AX foci were seen in WT ES cells, and comparatively, even fewer γH2AX foci in *Hdac6* overexpression ES cells than in WT ES cells (Fig. S1A and 1B). Also, *Hdac6* overexpression ES cells expressed lower γH2AX protein levels than did WT ES cells (Fig. S1C). These data show that high levels of Hdac6 can reduce DNA damage or induce more robust DNA repair.

Further, we compared telomere lengths of *Hdac6* overexpression ES cells with those of WT ES cells. Telomeres were longer in *Hdac6* overexpression ES cells than in WT ES cells shown as T/S ratio by qPCR method (Fig. S1D), telomere restriction fragment (TRF) analysis using conventional Southern blot (Fig. S1E) and quantitative fluorescence *in situ* hybridization (Q-FISH) (Fig. S1F). These data suggest that high expression levels of *Hdac6* can lead to telomere elongation in ES cells.

To look at down-stream genes regulated by overexpression of *Hdac6*, we performed the genome-wide expression analysis using Agilent Mouse Gene Expression Microarray, 8X60K chips (heatmap showing differential gene expression as 1.5 fold changes as cut-off, Fig. S1G). The microarray data was confirmed by QPCR analysis (Table S1). Genes for telomere elongation, e.g. *Tbx3* and *Zscan4* were up-regulated in *Hdac6* overexpression ES cells. Other prominently up-regulated genes included Trp73, Gadd45a, Sox17, Gata4 and Foxa2 (Fig. S1H and S1I). Sox17, Gata4 and Foxa2 are important in gonad and germ cell development, fertility and suppression of cell senescence. Genes involved in apoptosis, ubiquitin mediated proteolysis and DNA damage response also showed expression changes in *Hdac6* overexpression ES cells.

We tested whether Hdac6 influences epigenetic modification. H3K9me3 levels negatively regulate telomere lengths, but the histone acetylation levels positively regulate telomere lengths (Liu, 2017). H3ac and H3K9ac protein levels did not differ between *Hdac6* overexpression and WT ES cells, whereas H3K9me3 levels were reduced in *Hdac6* overexpression ES cells compared with WT ES cells (Fig. S1J). By ChIP-qPCR analysis, levels of H3K9ac at subtelomeres of selected chromosomes 7 and 13 were generally low, as expected, and did not differ between *Hdac6* overexpression and WT ES cells (Fig. S1K). However, H3K9me3 was enriched at subtelomeres in WT ES cells and the enrichment reduced in *Hdac6* overexpression ES cells (Fig. S1K). Moreover, H3K9me3 levels at *Zscan4* loci also were declined in *Hdac6* overexpression ES cells. These data show that Hdac6 can repress H3K9me3 and its enrichment at subtelomeres, associated with telomere elongation in ES cells.

*Hdac6* overexpression mice were generated from *Hdac6* overexpression ES cells (Wang et al., 2015). During breeding of *Hdac6* overexpression transgenic mice, we observed that *Hdac6* overexpression females in B6C3F1 background could still reproduce good number of pups by middle age, and even at the old age, in contrast to none or minimal number of pups produced from age-matched WT mice. Higher expression levels of *Hdac6* in the ovary of *Hdac6* transgenic mice than in WT mice were confirmed by immunohistochemistry (Fig. S2A), qPCR analysis (Fig. S2B), Western blot (Fig. S2C) and immunofluorescence microscopy (Fig. S2D). Hdac6 was expressed mostly in the cytoplasm and also in nuclei of cells in the cortex, oocytes, granulosa cells and corpus luteum (Fig. S2A and S2D). *Hdac6* overexpression female mice in hybrid background at the age of 12–16 months produced significantly larger litter size compared with age-matched WT mice (Fig. 1A). By backcross breeding of the hybrid F1 mice, we achieved a few litter of *Hdac6* overexpression inbred mice in C57BL/6 genetic background. *Hdac6* overexpression C57BL/6 females at the age of 10–12 month old still reproduced an average of four pups compared to none from age-matched WT mice (Fig. 1B). Higher levels of *Hdac6* are associated with extended reproductive age. Histological analysis of sections of ovaries collected from old mice revealed increased folliculogenesis and particularly secondary and antral follicles in *Hdac6* overexpression mice, compared with WT mice (Fig. 1C and 1D).

**Figure 1:**
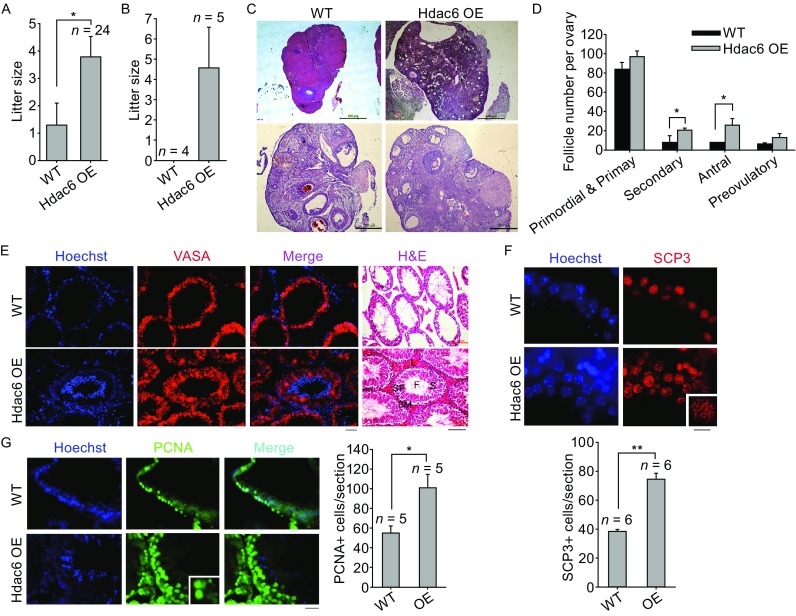
**Overexpression of** ***Hdac6*** **extends reproductive performance.** (A–D) Litter size and folliculogenesis in *Hdac6* overexpression and WT females. (A) Average litter size based on the number of old (age of 12 to 16 months) B6C3F1 female mice that were successfully mated, showing mating plugs. *P* < 0.05 by *t* test. (B) Average litter size produced from successfully mated old (about 10–12 months) C57BL/6 inbred females. *n* = number of successfully mated females. More old C57BL/6 mice were set to mate but failed from plugging. (C) Representative morphology of ovaries by hematoxylin and eosin (H&E) histology. Shown are ovaries from WT at 16-month-old and *Hdac6* OE mice at 19-month-old in B6C3F1 hybrid background. Scale bar = 500 μm. (D) Follicle development at the primordial and primary, secondary, antral, preovulatory stages. *P < 0.05. (E–G) Spermatogenesis and meiosis in old male mice. (E) Robust spermatogenesis in *Hdac6* OE mice shown by hematoxylin and eosin (H&E) histology of testis sections and immunofluorescence (IF) of VASA, indicative of germ cells, compared with much reduced spermatogenesis in WT mice at B6C3F1 hybrid background. Scale bars for IF = 20 μm; Scale bars for H&E = 100 μm. Testis section includes basement membrane (BM), Leydig cells (L), Sertoli cells (S), spermatogonia (SP) and filament (F). (F) Early meiocytes showing distinct synaptonemal complex protein-3 (SCP3) lateral filament structure at pachytene stage and quantification of average SCP3 positive cells per seminiferous tubule section. Inset, SCP3 lateral filaments at higher magnification. (G) Immunofluorescence of PCNA in testis from WT and *Hdac6* OE males and relative quantification of average PCNA positive cells per seminiferous tubule section. Scale bar = 20 μm (SCP3) or 50 μm (PCNA). *P < 0.05, **P < 0.01. *n* = number of tubule sections counted

Overexpression of *Hdac6* in the testis of *Hdac6* transgenic mice was shown by immunohistochemistry, immunofluorescence microscopy and qPCR analysis (Fig. S2E, S2F and S2G). Hdac6 expression was distributed in both nuclei and cytoplasm of various germ cells. Seminiferous tubules of old *Hdac6* overexpression males (19–21 month-old) were enriched with various germ cell types, while the age-matched WT controls showed much reduced number of germ cells in some seminiferous tubules (Fig. 1E). Abundant VASA positive cells were found in sections of seminiferous tubules in *Hdac6* overexpression testis, in contrast to some testis of WT mice (Fig. 1E), indicating that testis of old *Hdac6* overexpression males still actively undergo spermatogenesis and meiosis. Early meiocytes/spermatocytes are characterized by distinct synaptonemal complex protein-3 (SCP3) filament structure. Abundant PCNA+ proliferative germ cells and spermatocytes with homologous pairing marked by SCP3 filaments were found in testis of *Hdac6* overexpression mice, unlike age-matched WT mice (Fig. 1F and 1G). By TUNEL assay, fewer apoptotic cells were found in testis of *Hdac6* overexpression mice than in WT mice at the old age (Fig. S2H). These data indicate that germ cell attrition occurs in old males, but higher expression levels of *Hdac6* can reduce germ cell attrition and preserve spermatogenesis.

Follicle atresia and germ cell attrition could be related to autophagy. We did not find evident changes in the expression levels by qPCR of key genes for autophagy between *Hdac6* overexpression and WT mouse gonads (Fig. 2A). Autophagy-LC3B protein expression levels and distribution also did not differ between *Hdac6* overexpression and WT ES cells (Data not shown). We analyzed telomere lengths of somatic tails, and ovaries and testis from *Hdac6* overexpression mice in comparison with age-matched WT mice. Relative telomere lengths shown as T/S ratio were longer in tails and testis, and slightly longer in ovaries of *Hdac6* overexpression mice than those of age-matched WT mice (Fig. 2C). Interestingly, telomeres were longer in female tails than those of male tails in general. In males, telomeres were longer in testis than in somatic tails. Analysis of expression of genes important for telomere regulation showed that expression levels of telomerase genes *Tert* and *Terc*, telomerase activating gene *Sirt1*, and genes for meiosis recombination, *Spo11* or *Dmc1*, or cell senescence genes *p53*, *p16* or *mTOR* basically did not differ between *Hdac6* overexpression and WT gonads (Fig. 2B).

**Figure 2:**
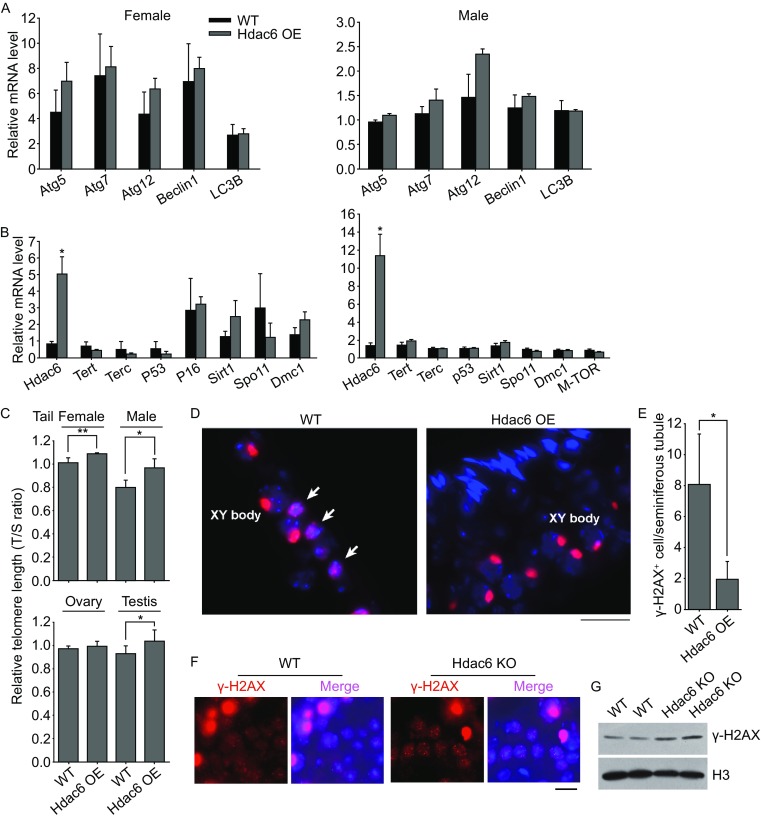
**Influences of** ***Hdac6*** **overexpression on telomere length maintenance and DNA damage response** ***in vivo***. (A) Relative mRNA levels by qPCR analysis of genes associated with autophagy of ovaries or testis of old WT and *Hdac6* OE females and males in B6C3F1 hybrid background. (B) Relative mRNA levels of genes related to telomere, meiosis and aging in ovaries or testis of old mice. WT mice were 15–16 months old and *Hdac6* OE mice 19 months old. (C) Relative telomere length shown as T/S ratio by qPCR analysis. For telomere analysis of tails, females were aged at 14–15 months, and males at 12–14 months (*n* = 4). For telomere analysis of gonads, female mice were 17–18 months old, and WT males at 15 months old, and *Hdac6* OE males at 19 months old. The data are expressed as mean ± SD (*n* = 4). (D and E) DNA damage shown by γH2AX foci is reduced in the seminiferous of *Hdac6* OE testis than in WT mice (19 month old). (D) Immunofluorescence of γH2AX indicated DNA damage with fragmented DNA (white arrows) and paired XY chromosomes at pachytene (indicated as XY body) in the seminiferous tubule. (E) Quantification of average number of γH2AX positive cells with fragmented DNA per seminiferous tubule of WT and *Hdac6* OE testis sections. (F and G) DNA damage is increased in *Hdac6* knockout (KO) mice compared with WT mice. (F) Immunofluorescence of γH2AX showing noticeable DNA damage foci in spermatocytes of *Hdac*6 KO testis but fewer foci in WT mice. Large spots in red are XY bodies, shown as in (D). (G) Western blot analysis of γH2AX protein levels. H3 served as a loading control. Scale bar = 20 μm. *P < 0.05, **P < 0.01

Furthermore, γH2AX positive cells with fragmented DNA foci, indicative of DNA damage response, were reduced in the testis of *Hdac6* overexpression mice compared with WT mice (Fig. 2D and 2E). γH2AX staining can detect DNA damage as well as the XY sex body for pairing at pachytene in spermatocytes that form unique large spot but not foci (Vasileva et al., 2013). On the contrary, *Hdac6* knockout mice exhibited increased DNA damage as evidenced by increased γH2AX foci in spermatocytes as well γH2AX protein levels in the testis, compared with WT mice at the same age (Fig. 2F and 2G).

Together, high levels of *Hdac6* can prolong reproductive life span in both males and females using transgenic mouse model. Furthermore, telomere maintenance, reduced DNA damage or increased DNA repair and less apoptosis together may contribute to the extended reproductive age by overexpression of *Hdac6*. The impact of Hdac6 on DNA damage response and telomere regulation could be indirect and the underlying regulatory mechanisms still warrant further investigation. Excitingly, ES cells with hyper-long telomeres generate healthier chimera mice displaying longer telomeres, reduced cell senescence and DNA damage with age, and better skin wound healing (Varela et al., 2016), further supporting the notion that longer telomeres from ES cells can be passed to their offspring that can achieve healthy aging.

Concerns may exist regarding potential risks of *Hdac6* overexpression in tumorigenesis. Inactivation or selective inhibition of *Hdac6* by inhibitors can greatly decrease cancer cell growth and proliferation (Lee et al., 2008), and the inhibition causes DNA damage (Namdar et al., 2010), in line with our observation here that overexpression of *Hdac6* reduces DNA damage, elongates telomeres, and increases cell proliferation. Presumably, longer telomeres and reduced DNA damage may reduce tumorigenesis in *Hdac6* overexpression mice. Further studies are required to investigate whether *Hdac6* overexpression mice may produce tumor at much older age or are more or less susceptible to tumor induction with age.

## Electronic supplementary material

Below is the link to the electronic supplementary material.

Supplementary material 1 (PDF 14498 kb)
