# Supplementary material for: Overexpression of Hdac6 extends reproductive lifespan in mice
Source: Protein Cell. 2017 Mar 1;8(5):360–4. doi: 10.1007/s13238-017-0375-9 (PMC5413599; doi:10.1007/s13238-017-0375-9)
Supplement: Supplementary file 1 — Supplementary material 1 (PDF 14498 kb) [file 13238_2017_375_MOESM1_ESM.pdf]

Supplementary Information

Methods
Tables S1-2
Figures S1-2

Methods

Mice and breeding

Hdac6 overexpression transgenic mice used in this study were generated previously (Wang et al., 2015), and age-matched WT mice served as aging controls (12-21 months old). Young male ICR mice at the age of 2-3 month old were used for breeding to test fertility of females. Late in the afternoon, one female mouse was placed in the cage of one male for mating. On the following morning, successfully mated female mice with plug were returned to their cages, and those without mating plugs were mated with a young male for another night. Females with no plugs for three successive days were not used for breeding experiments. The number of offspring within one week after delivery was recorded. *Hdac6* knockout (KO) mice were in 129/C57BL6 mixed genetic background, and were obtained from Tso-Pang Yao (Liu et al., 2015). All mouse experiments were carried out in accordance with the guidelines and regulations and approved by the Institutional Animal Care and Use Committee of Nankai University.

Serial sections of ovary or testis and follicle count

Ovaries or testis were collected and fixed by immersion in 4% paraformaldehyde for 8-24 h, and tissues were embedded with paraffin wax. Based on previous methods (Liu et al., 2013), serial sections (5 μm) from each ovary were aligned in order on glass microscope slides, stained with hematoxylin and eosin Y, and analyzed for the number of follicles in three or four different developmental stages in every fifth section with random start in the first five sections. The total number of follicles per ovary was calculated by cumulating the count of every fifth section throughout the whole ovaries.

The follicles were categorized into primordial and primary, secondary, antral and preovulatory, accordingly (Myers et al., 2004). Follicles were classified as primordial and primary if they contained an oocyte surrounded by a single layer of squamous or cuboidal granulosa cells. Follicles at an intermediate-stage also were scored in this group. Secondary follicles were identified as having more than one layer of granulosa cells with no visible antrum. Antral follicles possessed one or two small areas of follicular fluid (antrum) or a single large antral space. Preovulatory follicles had a rim of cumulus cells surrounding the oocyte.

Quantitative real-time PCR

Total RNA was isolated from tissues using RNeasy mini kit (Qiagen), and subject to cDNA synthesis using Moloney Murine Leukemia Virus Reverse Transcriptase (Invitrogen). PCR reactions were set up in duplicates using the FastStart Universal SYBR Green Master (Roche) and run on the Mastercycler® RealPlex2 real time PCR detection system (Eppendorf). At least two parallel samples were run for analysis of each gene. Primers were designed using the IDT DNA website and listed in Table S1. The final PCR reaction volume in 20μl contained 10μl SYBR Green PCR Master Mix, 1μl cDNA template, 2μl primer mixture and 7μl water. Thermal cycling was carried out with a 10 min denaturation step at 95 °C, followed by two-step cycles, 15s at 95°C and 1min at 60°C.

Immunocytochemistry and fluorescence microscopy

Briefly, after deparaffinizing, rehydrating and washing in 0.01 M PBS (pH 7.2–7.4), sections were incubated with 3% H2O2 for 10 min at room temperature to block endogenous peroxidase, subjected to high pressure antigen recovery sequentially in 0.01% citrate buffer (pH 6.0) for 3 min, incubated with blocking solution (5% goat serum and 1% BSA in PBS) for 2 h at room temperature, and then incubated with the diluted primary antibodies overnight at 4°C. Blocking solution without the primary antibody served as negative control. After washing with PBS, sections were incubated with appropriate secondary antibodies (Alexa Fluor® 568, 488 or 594, Invitrogen). The sections were then stained with 1 μg/ml Hoechst 33342 for 10 min to reveal nuclei, washed with PBS, mounted in Vectashield (H-1000, Vector Laboratories, Burlingame, CA, US) and photographed under a Zeiss Axio Imager Z1 (Carl Zeiss). The following primary antibodies were used for immunocytochemistry: VASA (ab13840, abcam, Cambridge, MA, US) (1:400), PCNA (SC25280, Santa Cruz, CA, US) (1:400), SCP3 (NB 300-232, Novus Biologicals, Littleton, CO, US) (1:400), HDAC6 (ab12173, Abcam) (1:100), β-tubulin (610153, BD)(1:200), LC3 (PM036, MBL)(1:200), γH2AX (05-636, Millipore) (1:200) or (4418-APC-020, TREVIGEN, 1:400).

Immunohistochemistry

Immunocytochemistry was performed based on the method described previously (Yuan et al., 2013).

Western blot

Cells were washed once in PBS, lysed in cell lysis buffer and boiled in SDS Sample Buffer at 99°C for 5 min; 20 μg total proteins of each cell extracts were resolved by 10% Bis-Tris SDS-PAGE and transferred to polyvinylidene difluoride membrane (PVDF, Millipore). Nonspecific binding was blocked by incubation in 5% non-fat milk or 5% BSA in TBST at 4 °C overnight. Blots were then probed with various primary antibodies by incubation overnight at 4°C in 5% skim milk in TBST. Immunoreactive bands were probed for 1-2 h at room

temperature with the appropriate horseradish peroxidase-conjugated secondary anti-Rabbit IgG –HRP (NA934V, GE Healthcare) or anti-mouse IgG-HRP (sc-2031, Santa Cruz). The protein bands were detected by ImmobilonTM Western Chemiluminescent HRP substrate (WBKLS0100, Millipore) and exposed to X-ray film. The following primary antibodies were used for western blot, γH2AX (05-636, Millipore) (1:1000), Hdac6 (ab12173, Abcam, Cambridge, MA, US) (1:500), H3 (ab1791, Abcam), H3K9ac (04-1003, Millipore), and H3K9me3 (ab8898, Abcam).

TUNEL assay

For detection of apoptosis cells, the *In situ* cell death detection kit was applied (Roche). Briefly, after deparaffinizing, rehydrating and washing in 0.01 M PBS (pH 7.2–7.4), sections were incubated in 0.1% TritonX-100 for 8-10 min. Then TUNEL reaction mixture was applied to the sections. After being stained with 1 μg/ml Hoechst33342 and mounted in Vectashield mounting medium (Vector Laboratories, CA, USA), sections were imaged using a Zeiss Axio Imager and apoptotic nuclei counted.

Telomere measurement

Telomere lengths were measured by qPCR (Callicott and Womack, 2006), and telomere Q-FISH following the protocol described (Dan et al., 2014). For qPCR of telomere length, the telomere signal was normalized to the signal from the single-copy gene (36B4) to generate a T/S ratio indicative of relative telomere length of the given sample. The TRF analysis by southern blot was performed using a commercial kit (TeloTAGGG Telomere Length Assay, catalog no. 12209136001, Roche Diagnostics), based on the method described previously (Blasco et al., 1997), with slight modifications. Cells were isolated and embedded in agarose plugs (Pulsed Field Certified Agarose, 162-0137, Bio-Rad) to let plugs containing 5×10^5 cells and treated with Proteinase K (PCR Grade, 03115879001, Roche Life Science). The plug was digested with MboI (R0147L, NEB) for 15 h and underwent electrophoresis through a 1% agarose gel in 1XTAE at 14°C for 16 h at 6 V/cm at an initial pulse for 1 s and ending in 12 s using the Bio-Rad CHEF DR-III pulse-field system.

ChIP-qPCR

ChIP-qPCR analysis of H3K9ac and H3K9me3 was performed as previously described (Dan et al., 2014). Briefly, $\sim 5 \times 10^7$ cells were fixed with 1% paraformaldehyde, lysed, and sonicated to achieve the majority of DNA fragments with 100-1000 bp. DNA fragments were then enriched by immunoprecipitation using 7 μg H3K9ac antibody (04-1003, Millipore) or 5 μg H3K9me3 antibody (ab8898, Abcam). The eluted protein:DNA complex was reverse-crosslinked at 65 °C overnight. DNA was recovered after RNase A and proteinase treatment. ChIP enriched DNA was analyzed by real-time qPCR and β-actin locus served controls. Primers are listed in Table S2.

Genome-wide gene expression by microarray analysis

ES cells were maintained on mitomycin-C treated mouse embryonic fibroblasts (feeders) in ES cell medium. Trizol extraction of total RNA was performed according to the manufacturer's instructions. Microarray was performed by CapitalBio Corporation (Beijing, China) using SurePrint G3 Mouse GE 8x60K Microarray (Agilent Technologies). The analysis was carried out according to the manufacturer's protocol, described in details on the website of CapitalBio (http://www.capitalbio.com). Only probe sets showing more than 1.5-fold change were retained in the final list. We then performed hierarchical clustering with the differentially expressed genes using cluster software (version 3), and the data has been deposited (GSE81710).

Statistical analysis

Statistical analyses were performed by ANOVA and means compared by Fisher’s protected least-significant difference (PLSD) using StatView software from SAS Institute Inc. (Cary, NC). P-value <0.05 or lower was considered statistically significant.

Blasco MA, Lee HW, Hande MP, Samper E, Lansdorp PM, DePinho RA, Greider CW (1997) Telomere shortening and tumor formation by mouse cells lacking telomerase RNA. Cell 91: 25-34.

Callicott RJ, Womack JE (2006) Real-time PCR assay for measurement of mouse telomeres. Comp Med 56: 17-22.

Dan J, Liu Y, Liu N, Chiourea M, Okuka M, Wu T, Ye X, Mou C, Wang L, Wang L*, et al.* (2014) Rif1 maintains telomere length homeostasis of ESCs by mediating heterochromatin silencing. Dev Cell 29: 7-19.

Liu M, Yin Y, Ye X, Zeng M, Zhao Q, Keefe DL, Liu L (2013) Resveratrol protects against age-associated infertility in mice. Hum Reprod 28: 707-717.

Liu N, Xiong Y, Li S, Ren Y, He Q, Gao S, Zhou J, Shui W (2015) New HDAC6-mediated deacetylation sites of tubulin in the mouse brain identified by quantitative mass spectrometry. Sci Rep 5: 16869.

Myers M, Britt KL, Wreford NG, Ebling FJ, Kerr JB (2004) Methods for quantifying follicular numbers within the mouse ovary. Reproduction 127: 569-580.

Wang D, Meng Q, Huo L, Yang M, Wang L, Chen X, Wang J, Li Z, Ye X, Liu N*, et al.* (2015) Overexpression of Hdac6 enhances resistance to virus infection in embryonic stem cells and in mice. Protein Cell 6: 152-156.

Yuan J, Zhang D, Wang L, Liu M, Mao J, Yin Y, Ye X, Liu N, Han J, Gao Y*, et al.* (2013) No evidence for neo-oogenesis may link to ovarian senescence in adult monkey. Stem Cells 31: 2538-2550.

Table S1. Primers for qPCR analysis

Genes	Forward	Reverse	Accession number
GAPDH	TCAACAGCAACTCCCACTCTTCCA	ACCACCCTGTTGCTGTAGCCGTAT	NM_008084.2
Tbx3	CCACCCGTTCCTCAATTTGAACAG	CGGAAGCCATTGATGGTAAAGCTGd	NM_011535
Zscan4	AAATGCCTTATGTCTGTTCCCTATG	TGTGGTAATTCCTCAGGTGACGAT	NM_001013765
Sox17	GATGCGGGATACGCCAGTG	CCACCACCTCGCCTTTCAC	NM_001289464.1
Bcl2l1	GACAAGGAGATGCAGGTATTGG	TCCCGTAGAGATCCACAAAAGT	NM_001289716.1
Ereg	CTGCCTCTTGGGTCTTGACG	GCGGTACAGTTATCCTCGGATTC	NM_007950.2
Apob	TTGGCAAACTGCATAGCATCC	TCAAATTGGGACTCTCCTTTAGC	NM_009693.2
Sox9	GAGCCGGATCTGAAGAGGGA	GCTTGACGTGTGGCTTGTTC	NM_011448.4
Bcl2	CGAGAAGAAGGGAGAATCACAGGA	AATCCGTAGGAATCCCAACCAGAG	NM_177410.2
Hdac6	CATTGCTGCTTTCCTGCACATCCT	TCCAGGGACAGAATCAACTTGCCT	NM_001130416.1
Tert	ACTGGTGGAGATCATCTTTCTGGG	ACCTGAGGAGTCTGACATATTGGC	NM_009354
Terc	CATTAGCTGTGGGTTCTGGTCT	TCCTGCGCTGACGTTTGTTT	NR_001579.1
P53	TGTTATGTGCACGTACTCTCCTCC	GTGCTGTGACTTCTTGTAGATGGC	NM_001127233.1
P16	CTAGCGTGTCTAGCATGTGGCTTT	TCTCATGCCATTCCTTTCCTGTCC	NM_001159589.1
Sirt1	AGCAACATCTCATGATTGGCACCG	TCTGCCACAGCGTCATATCATCCA	NM_019812.2
Spo11	GCTGGACAGCATCCTGAAGAGG	GGGTAAGTACACTCTGGACA	NM_012046
Dmc1	CAGATCCAGGAGCAACTATGA	CGATCCTCAGTTCTCCTCTT	NM_010059
Atg5	AAGTCTGTCCTTCCGCAGTC	TGAAGAAAGTTATCTGGGTAGCTCA	XM_011243108.1
Atg7	ATGCCAGGACACCCTGTGAACTTC	ACATCATTGCAGAAGTAGCAGCCA	XM_006506707.1
Atg12	AACAAAGAAATGGGCTGTGG	TTGCAGTAATGCAGGACCAG	NM_026217.3
Beclin1	TGAAATCAATGCTGCCTGGG	CCAGAACAGTATAACGGCAACTCC	NM_019584.3
LC3B	CTGGTGAATGGGCACAGCATG	CGTCCGCTGGTAACATCCCTT	NM_026160.4

Table S2. ChIP-qPCR primers

Primer	Forward	Reverse
β-actin	CGTGTGACAAAGCTAATGAGGCTG	CTAAGTTCAGTGTGCTGGGAGTCT
subtel_Ch7	GGGGGTCTTGATACAACTTCAGAA	TGCTGTTTCCCACCTTGACCTGTCC
subtel_Ch13	GCACACTTGGTGGGCTAAGAAGATG	TTAAATCCTGACCAAAATGCCTGGC
Zscan4 locus	GGGCTGAGCTGAGACTGATGACC	CACTTCAGCATCCACACTGGAGAGA

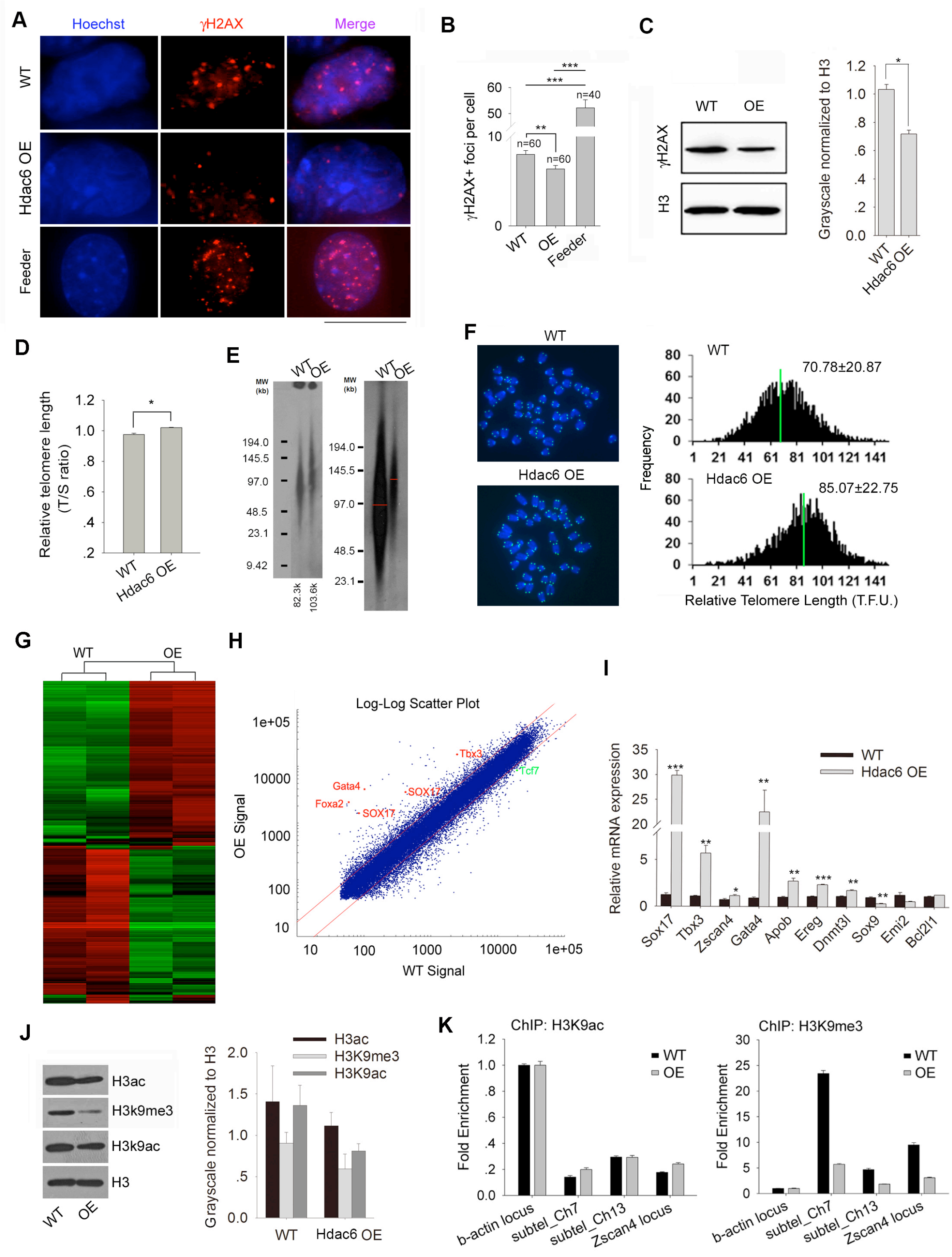

Figure S1. DNA damage response, telomere lengths and genome-wide expression of *Hdac6* overexpression (OE) and WT ES cells. (A) Immunofluorescence of γH2AX as a marker for DNA damage response. Feeder cells were treated with mitomycin C served as a positive control. Scale bar represents 10 μm. (B) Quantification of γH2AX positive foci per ES cell. The data are expressed as mean±SEM, **P<0.01, n=number of cells counted. (C) Protein levels of γH2AX by Western blot. Right panel, relative γH2AX protein levels shown as grayscale using Image J software. *P<0.05. (D-F) Telomere lengths of *Hdac6* overexpression and WT ES cells. (D) Relative

telomere length shown as T/S ratio by qPCR in *Hdac6* OE and WT ES cells. The data are expressed as mean±SEM, *P<0.05. (E) Telomere length distribution shown as TRF by Southern blot analysis. Two repeated experiments. (F) Representative images showing telomeres of *Hdac6* OE and WT ES cells by Q-FISH. Right panel, Histogram showing relative telomere length distribution analyzed by TFL-TELO software. TFU, arbitrary telomere fluorescence unit. Green line indicates median telomere length from at least 15 spreads per group. (G) Heatmap showing differentially expressed genes between WT and *Hdac6* OE ES cells by microarray. The green color indicates down-regulated genes, while the red color indicates up-regulated genes. FC(abs)>1.5 and p-value≤5% were used as analysis standard. (H) Scatter plot showing differentially expressed genes between WT and *Hdac6* OE ES cells. Red, up-regulated genes; green, down-regulated genes. (I) QPCR analysis of selected genes showing transcriptional changes in the microarray. RNA was purified from WT and *Hdac6* OE ES cells at the same passage (P26) for microarray. Relative expression was shown as fold differences relative to GAPDH. The data are expressed as mean±SEM (*P<0.05, **P<0.01, ***P<0.001). (J) Western blot analysis of H3ac, H3K9ac and H3K9me3 protein levels in *Hdac6* OE and WT ES cells. H3 served as a loading control. Right panel, Relative protein levels shown as gray scale using Image J software. (K) ChIP-qPCR assay of H3K9ac or H3K9me3 levels at sub-telomeres of chr7, chr13 and proximal regions of Zscan4 promoter in *Hdac6* OE and WT ES cells. The β-actin locus served as control. Two repeats.

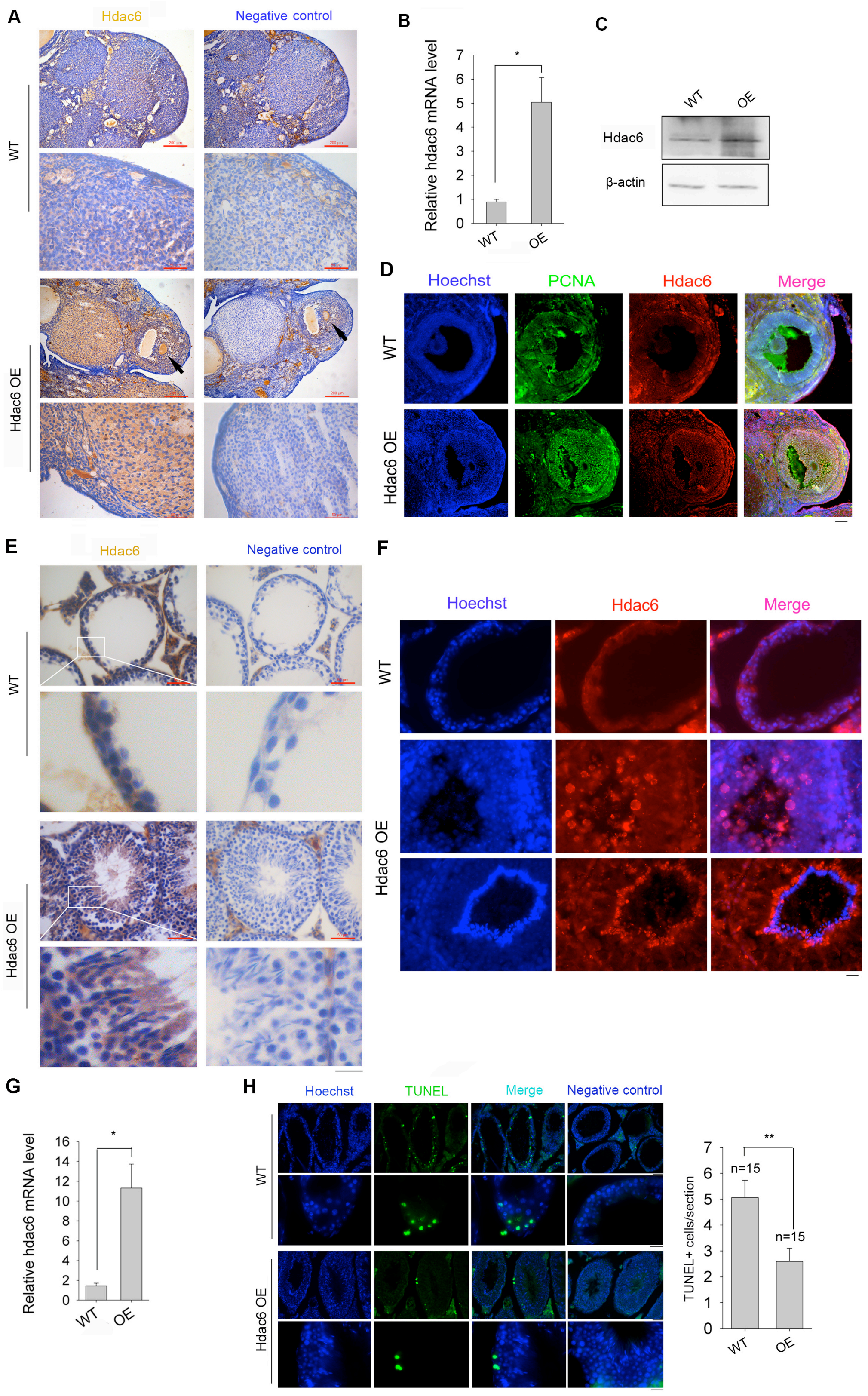

Figure S2. Expression of Hdac6 in WT and *Hdac6* OE mice. (A) Immunohistochemistry by DAB reaction of ovaries. Shown are representative images from 18-month-old Hdac6 OE mouse and 14-month-old WT mouse. Scale bars for upper panel = 200 μm and lower panel = 50 μm. Black arrow, indicates Hdac6 brownish staining in oocyte cytoplasm, but not in negative

controls without adding the Hdac6 antibody. (B) Relative mRNA levels by qPCR of *Hdac6* in ovaries. The data are expressed as mean±SEM, *P<0.05. (C) Western blot analysis of Hdac6 protein levels. (D) Immunofluorescence co-staining of PCNA and Hdac6 in ovaries of WT and *Hdac6* OE females. Proliferative granulose cells are shown as PCNA positive cells. Both WT and Hdac6 OE female mice were at the age of 16-17 months. (E-H) Expression of Hdac6 by immunohistochemistry, immunocytochemistry and qPCR, and apoptosis assay in old WT and *Hdac6* overexpression (OE) males in B6C3F1 hybrid background. (E) Expression of Hdac6 revealed by immunohistochemistry with DAB staining. At least three mice per group were used in DAB staining. Scale bars = 50 μm. (F) Immunofluorescence microscopy images showing relatively higher expression levels of Hdac6 in Hdac6 OE males than in WT males at similar old age. (G) mRNA levels of Hdac6 by qPCR in the old mice. (H) Apoptosis by TUNEL assay, and quantification of average TUNEL positive cells per tubule section. At least three mice per group were used in statistic analysis. Scale bars for upper panel = 50 μm and lower panel = 20 μm. n= number of tubule sections counted. mean±SEM, *P<0.05, **P<0.01.
